# Aire in Autoimmunity

**DOI:** 10.1146/annurev-immunol-090222-101050

**Published:** 2024-06-14

**Authors:** Corey N. Miller, Michael R. Waterfield, James M. Gardner, Mark S. Anderson

**Affiliations:** 1 Diabetes Center, University of California, San Francisco, California, USA; 2 Department of Medicine, University of California, San Francisco, California, USA; 3 Department of Pediatrics, University of California, San Francisco, California, USA; 4 Department of Surgery, University of California, San Francisco, California, USA

**Keywords:** Aire, autoimmunity, immune tolerance, mimetic cells, Janus cells, eTACs

## Abstract

The role of the autoimmune regulator (Aire) in central immune tolerance and thymic self-representation was first described more than 20 years ago, but fascinating new insights into its biology continue to emerge, particularly in the era of advanced single-cell genomics. We briefly describe the role of human genetics in the discovery of Aire, as well as insights into its function gained from genotype–phenotype correlations and the spectrum of Aire-associated autoimmunity—including insights from patients with Aire mutations with broad and diverse implications for human health. We then highlight emerging trends in Aire biology, focusing on three topic areas. First, we discuss medullary thymic epithelial diversity and the role of Aire in thymic epithelial development. Second, we highlight recent developments regarding the molecular mechanisms of Aire and its binding partners. Finally, we describe the rapidly evolving biology of the identity and function of extrathymic Aire-expressing cells (eTACs), and a novel eTAC subset called Janus cells, as well as their potential roles in immune homeostasis.

## 1. INTRODUCTION

Autoimmune diseases represent a significant health burden, affecting more than 400 million people worldwide, and arise in part through a breakdown in normal immune tolerance. A key to improving our understanding and advancing our treatment of these diseases is to unravel the defects in immune tolerance present in affected individuals. Immune tolerance is controlled by a multilayered system that includes key cell populations, regulatory molecules, suppressive cytokines, and complex immune cell selection processes. An approach to identifying these important components has been genetic analyses of persons affected by autoimmunity. Indeed, over the last 25 years we have witnessed a revolution in identifying common variants that contribute to autoimmune susceptibility, with a strong influence of certain *HLA* alleles in many diseases. An alternative approach has been to examine patients with monogenic forms of autoimmunity that harbor rare genetic variants or mutations. Here, a number of critical genes that help promote immune tolerance have been identified, including *FOXP3*, *IL2RA*, *CTLA4*, and *AIRE*. In this review, we focus on the autoimmune regulator (AIRE) gene and provide an update on recent insights into its key roles in promoting immune tolerance.

## 2. APS1 AND AIRE: CLINICAL OBSERVATIONS PROVIDING THE PATH TO THE GENE AND INSIGHTS INTO AUTOIMMUNITY

The monogenic syndrome autoimmune polyglandular syndrome type 1 (APS1) was originally described in 1943 (1). Typically, patients develop a panoply of organ-specific autoimmune diseases over time that emerge during childhood. Common clinical features include the classic triad of hypoparathyroidism, Addison’s disease, and intermittent episodes of mucocutaneous candidiasis (2). In addition, most patients develop other autoimmune features that can involve a range of organs. The syndrome has a classic autosomal recessive inheritance pattern, with siblings occasionally affected. For this reason, extended families were genetically characterized during the 1990s, and the defective gene was identified through extensive positional cloning and termed the autoimmune regulator (AIRE) (3, 4).

**Figure 1:**
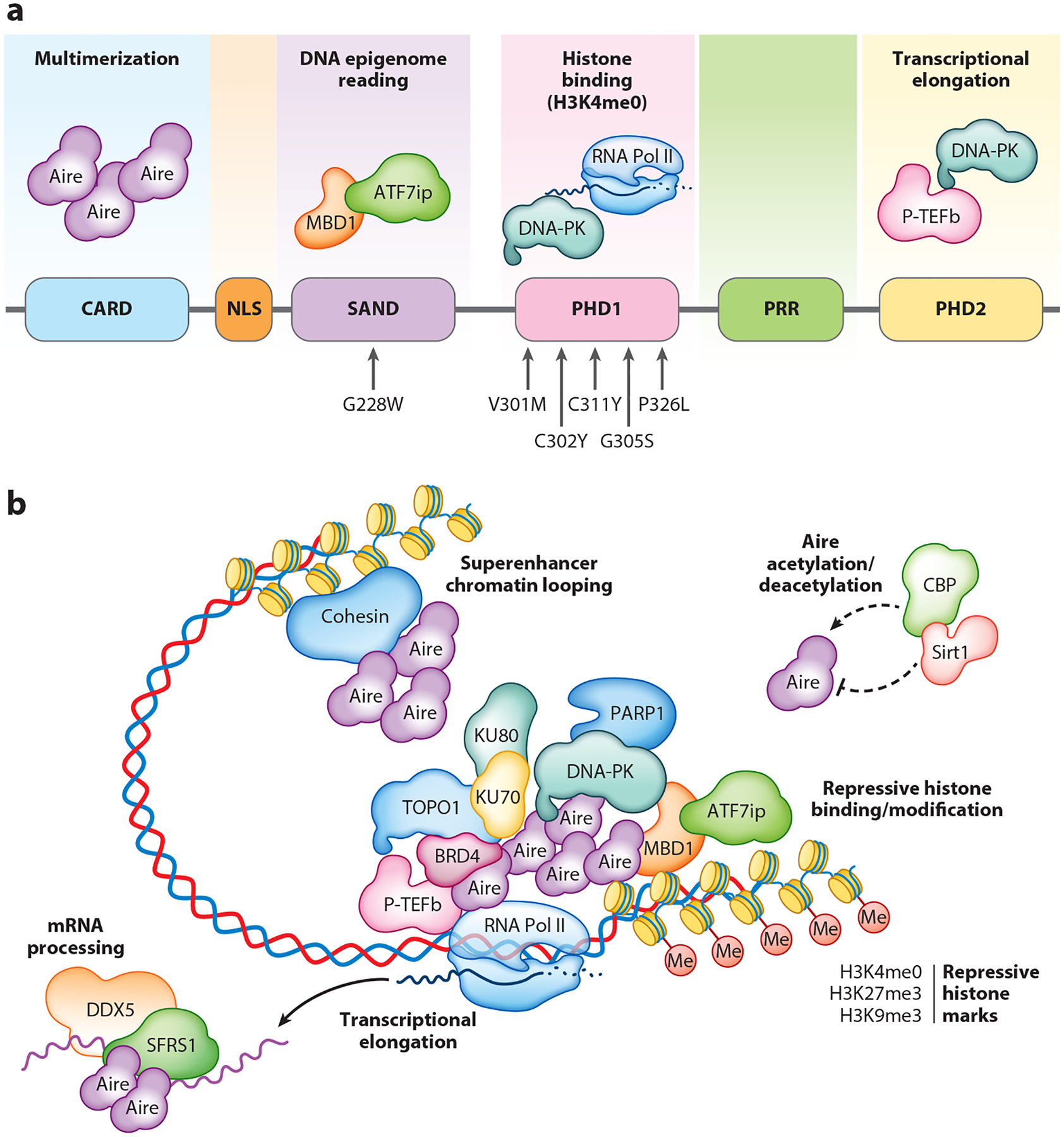
Domains and binding partners of Aire. (*a*) Schematic of the AIRE protein and its conserved domains. The human AIRE protein is 545 amino acids long and has five major conserved domains: CARD, SAND, PHD1, PRR, and PHD2. CARD is involved in the multimerization of AIRE, while PHD1 is a zinc-finger domain that has specificity for binding to and recognizing H3K4Me0 marks on chromatin (167). APS1 is classically autosomal recessive, and deleterious mutations are described across the length of the protein. Dominant mutations in AIRE can also occur through missense mutations downstream of CARD that cluster in the SAND domain and PHD1 and confer a hypomorphic effect on AIRE function. (*b*) Binding partners and molecular functions (and regulators) of the Aire complex, highlighting roles in histone binding and modification, transcriptional elongation and mRNA processing, and superenhancer binding and chromatin looping. Abbreviations: APS1, autoimmune polyglandular syndrome type 1; CARD, caspase recruitment domain; CBP, CREB-binding protein; NLS, nuclear localization sequence; P-TEFb, positive transcription elongation factor b; PHD, plant homeodomain; PRR, proline-rich region; SAND, Sp100, AIRE1, NucP41/75, DEAF1.

The *AIRE* gene encodes a 545-amino-acid protein that has at least four distinct domains, and most affected patients harbor biallelic mutations that likely cripple AIRE function. Among the different domains are the caspase recruitment domain (CARD), the SAND (Sp100, AIRE1, NucP41/75, DEAF1) domain, and plant homeodomains 1 and 2 (PHD1 and PHD2) (Figure 1*a*). AIRE also harbors a nuclear localization signal sequence and localizes to nuclear speckles (5, 6). AIRE forms homomultimers through its CARD, and many missense mutations cluster in this region in patients with APS1 (7). Because AIRE can self-multimerize through CARD, it is possible that certain downstream missense mutations can confer dominant interference. Indeed, we have now come to appreciate that mutations in *AIRE* that cluster in the SAND domain and PHD1 can lead to an autosomal dominant pattern of inheritance of autoimmunity, with typically less severe features (5, 8, 9). Finally, more common variants in AIRE have been linked to susceptibility to Addison’s disease (10). Thus, the discovery of AIRE as a key checkpoint on immune tolerance epitomizes the phrase often attributed to William Harvey: “Medicine is the great tutor of biology.” Only through the careful study of patients affected by such a rare autoimmune syndrome have we gleaned these fundamental mechanistic insights into immune homeostasis, with their diverse implications for not only APS1 but also more common forms of autoimmunity and human health.

Another important clue into the activity of AIRE in immune tolerance came from determining its expression pattern, which is localized to medullary thymic epithelial cells (mTECs) and, as demonstrated more recently, to rare hematopoietic populations in peripheral lymphoid tissues termed extrathymic Aire-expressing cells (eTACs; discussed further in Section 5). Within the thymus, Aire-expressing mTECs have the unique property of ectopically or promiscuously expressing a wide array of self-antigens that otherwise are expressed in restricted areas of the body (11). We now appreciate that expression of a significant number of these otherwise tissue-specific antigens (TSAs) in mTECs is controlled at least in part by AIRE (12). Thus, AIRE is a unique transcriptional regulator that helps promote the display of the peripheral self, thereby enabling thymocyte selection against TSAs and enforcement of central T cell tolerance. We have also come to appreciate that a fraction of Aire-expressing cells further differentiate into multiple post-Aire subtypes that mimic a range of differentiated barrier epithelial cell types in peripheral tissues, dubbed mimetic cells (13–16). This review addresses these important recent insights into the molecular and developmental mechanisms by which AIRE and other factors control this process in the thymus. Finally, because of the inherent complexity of thymic selection and its relative inaccessibility for study, much of the foundational research for this biology has been performed in mice, but recent studies using single-cell genomic approaches in human thymus now support similar models of TEC differentiation and diversity (17–19).

As mentioned above, APS1 patients typically develop an array of autoimmune features, often associated with organ-specific autoantibodies. Interestingly, autoantibodies in APS1 patients are frequently high affinity, and serum from these patients has been a rich resource for the identification of novel autoantibody specificities that can explain clinical features of the disease. For example, NALP5 autoantibodies were identified as being strongly associated with hypoparathyroidism, and NALP5 demonstrates some specificity for the parathyroid gland (20). Likewise, APS1 patients frequently develop autoantibodies to certain cytokine families, including IL-17A, IL-17F, IL-22 (21, 22), and type 1 interferons, particularly type 1 IFN-α and -ω family members (23). Autoantibodies against T helper 17 (Th17)-related cytokines may partly explain the susceptibility to candidiasis in these patients, while type 1 interferon autoantibodies confer susceptibility to viral infections including severe COVID-19 (see the sidebar titled Aire and APS1 Offer Key Insights into Severe COVID-19 Susceptibility).

Novel platforms have recently been developed to widely test for autoantibodies in printed arrays as well as in yeast and phage-display libraries that harbor thousands of targets tiling the entire proteome (24–27). The analysis of serum from APS1 patients through the use of such platforms has led to the identification of a wide variety of new autoimmune targets. Autoantibodies to the transcription factor RFX6 were identified in numerous APS1 patients and correlated strongly with the development of intestinal dysfunction and diarrhea (27). Another recently identified target in APS1 is Perilipin 1 (PLIN1), which was found as an autoantibody target in an APS1 patient with the unusual feature of acquired generalized lipodystrophy (28). PLIN1, a lipid droplet protein that helps control fatty acid release, is highly expressed in subcutaneous fat. Loss of PLIN1 in both mice and humans leads to the development of a loss of subcutaneous fat, fatty liver, and insulin resistance, all features of lipodystrophy (29, 30). Interestingly, it has long been appreciated that an acquired form of generalized lipodystrophy is associated with other autoimmune conditions (31). The identification of PLIN1 autoantibodies in a single APS1 patient with lipodystrophy led to its subsequent testing in a large pool of acquired generalized lipodystrophy patients, and indeed, more than 35% of such patients harbor these same autoantibodies (28, 32). This finding lends credence to the idea that deeply studying autoimmunity in patients with a rare disease like APS1 can lead to a broader understanding of immune homeostasis with applications in diverse clinical settings.

## 3. AIRE AND THYMIC EPITHELIAL DEVELOPMENT AND DIFFERENTIATION

### 3.1. Origins of Medullary Thymic Epithelial Cell Subsets

While Aire has historically been thought of as a purely transcriptional regulator of TSA expression in terminally differentiated mTECs, recent research has revealed a more complicated role in mTEC biology, particularly as it relates to mTEC development. Multiple recent reviews devoted to the general topic of TEC development provide excellent, comprehensive overviews (46, 47); here, we provide a brief background and focus on Aire expression as a seminal developmental event during steady-state mTEC differentiation, including Aire’s influence on terminally differentiated mimetic subsets.

**Figure 2:**
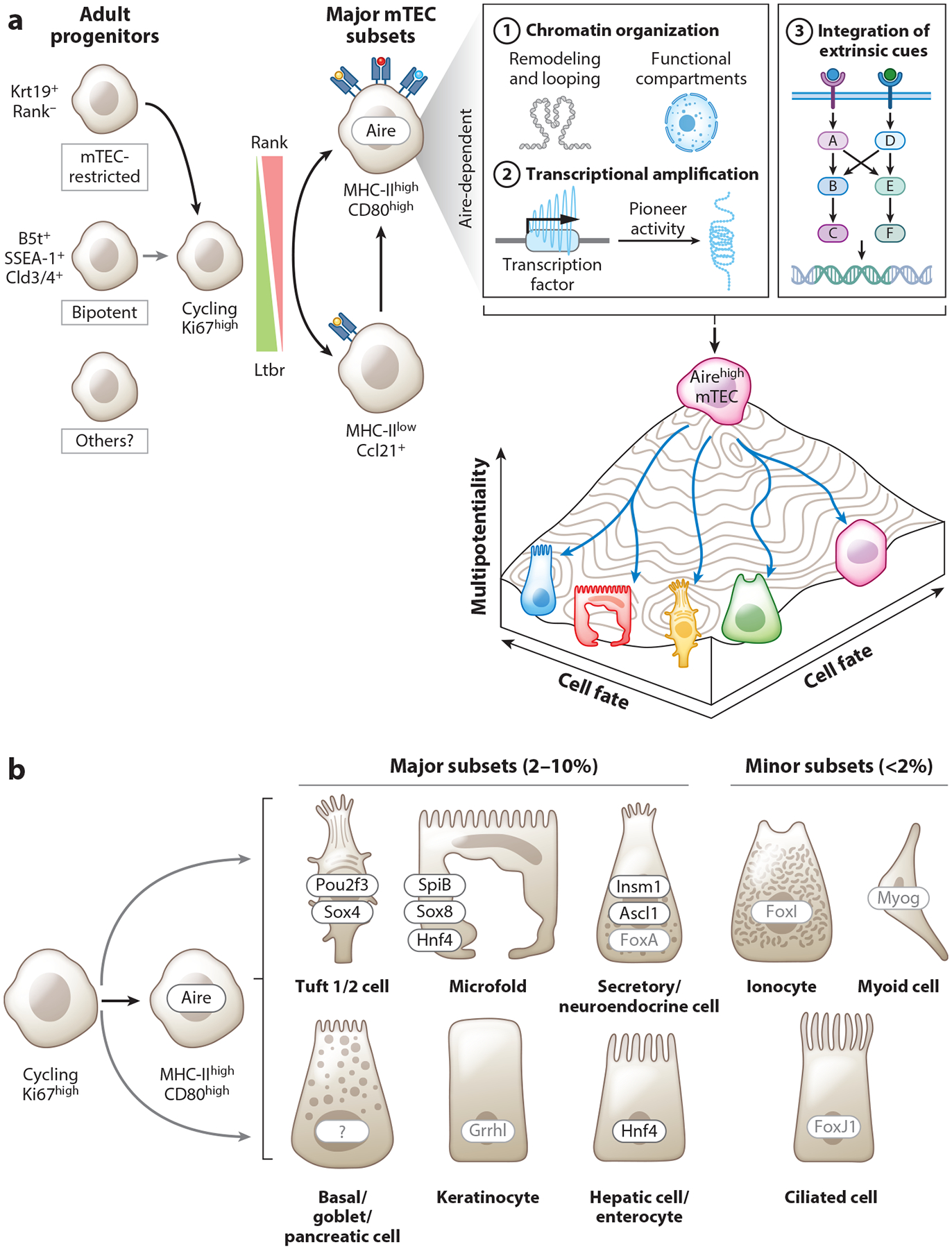
mTEC differentiation and relationship to Aire expression. (*a*) TEC development proceeds from multiple progenitor populations with varying commitment toward the mTEC lineage. Dominant Rank signaling induces the Aire^+^MHC-II^high^CD80^high^ stage of development, characterized by high transcriptional complexity and a high capacity for antigen presentation. Within such cells, Aire-mediated transcriptional activation likely contributes to a metastable transitory state that amplifies access to multilineage epithelial differentiation. Possible mechanisms include direct effects on chromatin looping and remodeling, organization of or recruitment to functional nuclear compartments for transcription and mRNA splicing, and amplification of transcriptional noise. The expression of additional transcriptional regulators capable of binding closed chromatin (i.e., pioneer factors) is likely to be self-reinforcing and guide fate determination, together with local availability of extrinsic cues, such as cytokines and metabolic products. (*b*) Mimetic subsets and their relative abundance in scRNA-seq data sets. Lineage-defining transcription factors with orthogonal experimental evidence are shown in black font, and those proposed on the basis of bioinformatic analyses alone are shown in gray font. Abbreviations: mTEC, medullary thymic epithelial cell; scRNA-seq, single-cell RNA sequencing; TEC, thymic epithelial cell.

The identity of the thymic epithelial stem and progenitor cells (TEPCs) that maintain the cortical TEC (cTEC) and mTEC compartments has been a subject of long-standing interest. Unlike many peripheral epithelial tissues, where clearly defined adult stem populations have now been described, multiple putative progenitor populations with varying degrees of lineage potential have been proposed in the thymus. For the thymic medulla, there is an emerging consensus that late embryonic and postnatal mTEC homeostasis transitions from a bipotent (cTEC and mTEC) progenitor toward lineage-biased, mTEC progenitors (48–51) (Figure 2*a*). Within cells definitively committed to the mTEC lineage, the precise developmental relationships also remain uncertain. Until recently, mTECs (minimally defined as CD45^−^Epcam^+^Ly51^−^ by flow cytometry) were considered to consist of relatively few bulk populations, defined largely by surface MHC-II levels and several additional markers (e.g., Aire, CD80). Progenitors and less mature mTECs were shown to reside within the MHC-II^low^ (mTEC^low^) compartment and give rise to Aire- and CD80-expressing MHC-II^high^ cells (mTEC^high^) (48). However, the application of single-cell RNA-sequencing (scRNA-seq) and other high-dimensional methodologies to the thymus has greatly expanded and fundamentally transformed our understanding of mTEC heterogeneity, revealing unexpected subsets and offering a window into novel biology (discussed further in Section 3.3, below) (13, 14).

With respect to early lineage relationships, we used scRNA-seq to observe that a substantial fraction of MHC-II^low^ mTECs express the Ccr7 ligand Ccl21, in line with previous reports, and cluster separately from the bulk population of Aire-expressing mTECs (52). Interestingly, a separate and prominent cluster of cycling mTECs was also found, and these cells expressed low levels of Ccl21 alone, Aire alone, or both Ccl21 and Aire transcripts, suggesting a possible branch point feeding more stable populations of MHC-II^low^Ccl21-expressing or MHC-II^high^Aire-expressing cells (52). Such a branching model contrasts with the linear, unidirectional model that has generally been favored, in which most or all pre-Aire-expressing MHC-II^low^ cells differentiate into Aire-expressing MHC-II^high^ cells. Evidence for the latter has leveraged genetic lineage-tracing systems, and these models support the conclusion that a considerable fraction of Aire-expressing mTECs derive from cells that have previously expressed Ccl21 (53, 54).

Other recent scRNA-seq descriptions of proliferating TECs have reached various conclusions regarding the developmental dynamics between Ccl21^+^- and Aire-expressing mTECs, generally by employing bioinformatic tools such as RNA velocity or pseudotime (55, 56). None of the data presented to date definitively address whether some or most MHCII^low^Ccl21-expressing mTECs represent a stable, postmitotic population or whether they uniformly maintain the capacity for further maturation.

Importantly, the clustering of cells into a cycling population is partially driven by transcriptomic similarities related to active cell division, and such cells should not be assumed to originate from a single microanatomical niche, such as the corticomedullary junction. Cycling mTECs may derive from one, several, or diverse spatial niches with different access to extrinsic inductive signals and, therefore, different tendencies to give rise to Ccl21^high^, Aire^high^, and other terminally differentiated mTEC lineages (57).

Recently, researchers identified a population of CD9^+^Krt19^+^MHC-II^−/low^ thymic epithelial cells (TECs) that lack Ccl21 and Aire expression but preferentially give rise to these major mTEC subsets in both embryonic and adult thymus, suggesting that Krt19 may mark an important mTEC progenitor preceding cycling mTECs (58). While the specific signals maintaining TEPCs remain elusive, evidence points to Notch signaling as a key early switch and Fgf7 as a possible maintenance factor (59–62). Ultimately, defining the precise lineage relationships and developmental dynamics between mTEC populations is likely to require novel lineage-tracing approaches, such as high-resolution CRISPR-Cas9 scarring systems, an early version of which has been applied to the thymus with some success (62). Future versions of these systems are expected to generate sufficient barcode diversity to resolve precursor product relationships in complex tissues, and their use in the thymus is likely to enable a rapid increase in understanding of the lineage relationships among TEPCs, Ccl21^+^ cells, Aire-expressing cells, and the rapidly expanding universe of terminally differentiated mTEC populations (63).

### 3.2. Induction of the Aire Lineage

Regardless of the identity of the mTEC progenitor(s), approximately 40% of mTECs in adult steady state murine thymus are marked by high levels of MHC-II and Aire protein expression. It is now well established that noncanonical and canonical NF-κB signaling downstream of TNF superfamily receptors, and especially Rank, is essential for the development and maintenance of mTECs and the Aire-expressing compartment (50, 64–67) (Figure 1*a*). While there are multiple distinct cellular sources of TNF superfamily ligands in the thymus, single-positive CD4^+^ thymocytes are an especially potent source of Rankl (65). Downstream binding of NF-κB to the Aire promoter is mediated, at least in part, by a proximal highly conserved noncoding sequence termed CNS1 (68, 69). Within specific niches, diverse hematopoietic subsets, including thymic innate lymphoid cells (ILCs), invariant T cell subsets, and B cells, make important though partially redundant contributions to the balance of available lymphotoxin, LIGHT, CD40L, or RANKL ligands (70, 71). In this way, the integration of locally dominant signals, such as lymphotoxins, are thought to guide specific phases of mTEC developmental or lineage trajectories, such as for Ccl21^+^ subset maturation and thymic tuft cell development (72, 73).

Although NF-κB signaling is required for Aire expression in mTECs, additional transcriptional regulators and molecular mechanisms have started to be elucidated. A careful examination of Aire promoter function has revealed both epigenetic modifications associated with Aire expression and evidence for direct transcriptional activation by coordinated binding of Irf4, Irf8, Tbx21, and Tcf7 at the Aire transcriptional start site (74). An improved understanding of the transcriptional machinery driving Aire expression, and especially heterogeneity in the availability of such factors during the differentiation of individual mTECs, is likely to inform not only regulation of the Aire locus but also the broader regulatory landscape that is inherited by nascent Aire-expressing cells and upon which Aire then acts.

### 3.3. Differentiation Beyond Aire: Back to the Future

Establishment of Aire expression in MHC-II^high^ mTECs profoundly alters the transcriptome, and this aggressive transcriptional regulation has become foundational to our understanding of self-representation in mTECs. Prior to the discovery of Aire, however, there were existing ideas about how the thymic epithelium might orchestrate proteomic coverage for negative selection. One idea, eventually dubbed progressive restriction, was based on long-standing light and electron microscopic observations of diverse epithelial cell morphologies within the thymus (75). In this model, immature mTECs progressively differentiated toward a mosaic of diverse peripheral epithelial lineages, presumably through ordered developmental pathways. Here, transcriptional activation induced by Aire was thought to support a permissive cellular state for downstream developmental diversification, and this idea was perhaps most championed by Farr and colleagues (76–78). Under this progressive restriction model, Aire-expressing mTECs were akin to stemlike progenitors that gave rise to high cellular diversity in daughter populations but were not necessarily the source of transcriptional diversity themselves.

Another idea was that mTECs might feature leaky or promiscuous gene expression that slowly accumulated transcriptional diversity but was not biologically ordered. In this model, called terminal differentiation by its proponents, the discovery of Aire provided a molecular mechanism for stochastic gene expression (79, 80). The key distinction, and the origin of the moniker, was the assertion that the accumulation of disordered gene expression would promote cell death, making Aire expression a terminal event. Indeed, in a very early example of single-cell transcriptional analysis (81), researchers used high-sensitivity polymerase chain reaction to examine Aire-dependent gene expression in single, sorted Aire^+^MHC-II^high^ mTECs and found TSA expression to be probabilistic and stochastic, without clear signs of preferential coexpression of particular extrathymic epithelial lineage markers. A contemporaneous study by the same group found MHC-II^high^ mTECs to be postmitotic (82). Together, these reports appeared to heavily favor the terminal differentiation model, but the interpretation of the findings was based on two limiting assumptions. First, by analogy to dendritic cell biology, high levels of MHC-II and CD80 expression were assumed to be rarely reversed steps in cellular maturation, leading to a focus on MHC-II^high^ mTECs. Second, by analogy to stem cell biology, progressive restriction was assumed to imply a wave of mitotic cell division by Aire-expressing mTECs undergoing epithelial transdifferentiation. The possibility that biological order might emerge in a subset of post-Aire-expressing mTECs was lost in dueling ideologies, and the discovery of these cells would require the development of novel tools.

Definitive evidence of mTEC survival after the expression of Aire, or a “post-Aire” stage of mTEC development, began to emerge with the engineering of various Aire reporter and Aire lineage-tracing mouse lines (83–85). These tools allowed identification of mTECs that had previously passed through an Aire-expressing stage; critically, such cells were found to exist and to be MHC-II^low^. Reporters provided insight into the kinetics and complexity of mTEC development— with overall mTEC half-life estimated to be 12–14 days and the post-Aire half-life estimated to be 7–8 days—and showed definitively that the MHC-II^low^ compartment contained not only immature TECs but also a mixture of precursor and product populations.

By combining active Aire reporting and inducible Aire lineage-tracing alleles, we sorted definitive post-Aire mTECs without contamination from other subsets for bulk transcriptomic analysis (13). Remarkably, multiple distinct terminally differentiated epithelial gene signatures were recognizable within the post-Aire compartment, including those of epithelial cornification and, unexpectedly, peripheral tuft cells. A contemporaneous study using plate-based scRNA-seq also identified the peripheral tuft gene signature in approximately 10% of sorted mTECs (14). Together, these findings clearly aligned with the progressive restriction model and demonstrated that at least some post-Aire mTECs differentiate into highly specialized barrier epithelial cells. Shortly thereafter, droplet-based scRNA-seq was rapidly extended to human thymus, where tuft cell, cornified keratinocyte, ciliated cell, neuroendocrine cell, ionocyte, and myoepithelial gene signatures were described (17, 18). However, because these studies focused on human thymus, it was unclear whether this striking epithelial heterogeneity emerges from Aire-expressing mTECs or depends on Aire function.

Recently, significant progress has been made through the analysis of murine mTECs by combined single-cell assay for transposase-accessible chromatin sequencing (scATAC-seq), scRNA-seq, and Aire lineage tracing and sorting for post-Aire cells (16). This research enabled the highest-resolution description of terminally differentiated mTEC subsets to date and defined significant and novel TEC lineage heterogeneity (e.g., goblet-like, microfold-like, and enterocyte-like TECs) (Figure 2*b*). The authors of this study (16) dubbed these terminally differentiated mTECs collectively as “mimetic cells” because they were observed to transcriptionally mimic discrete populations in the peripheral epithelium. Importantly, through multiple cutting-edge approaches, they organized mimetic subsets around lineage-defining transcription factors that were shared with peripheral epithelial counterparts. Therefore, a particular strength of this study was the confirmation of biological order as a generalizable feature of post-Aire mTEC development, and the authors have now extended this idea through a focused examination of specific developmental pathways (86). Interestingly, while mimetic cell development depended on prior Aire expression, mimetic differentiation persisted in Aire^−/−^ thymi, and transcriptional changes within a given subset were surprisingly minimal. Therefore, Aire was found to amplify terminal differentiation rather than be strictly required for it, a finding that aligns with both earlier and subsequent research (13, 87).

How Aire contributes to mimetic cell development is a fascinating question. Given its role as a multifaceted transcriptional activator, Aire may increase the likelihood of lineage diversification by amplifying inherited metastable gene regulatory networks and transcriptional noise until self-reinforcing transcriptional patterns establish new stable minimums of terminal differentiation (88). In such a model, the balance of mimetic subsets would be guided by inherited regulatory machinery and heterogeneity across the chromatin landscape from pre-Aire progenitors and the integration of local extrinsic signals during active Aire expression (Figure 2*a*). How these variables might intersect with Aire-mediated chromatin and genome remodeling around superenhancers and the formation of functional nuclear condensates are interesting areas for further investigation. Rapidly evolving technologies for mapping three-dimensional chromatin structure at the single-cell level may be useful for gaining insight into the “rules” governing Aire’s localization within individual nuclei (89). Perhaps presciently, Farr and colleagues (77) reported Aire-dependent mTEC expression of transcription factors in association with embryonic plasticity of stem progenitor cells, including Oct4, Sox2, and Nanog. Further studies will be needed to understand how to integrate this finding with our current understanding of the molecular mechanisms of Aire and the newly revived mimetic model, but it suggests the possibility that expression of transcription factors with so-called pioneering activity and an ability to bind DNA packaged into closed chromatin may facilitate or precede accessibility to some mimetic outcomes (90).

Looking forward, the recognition of biologically ordered mimetic differentiation in mTECs, often following the expression of Aire, raises far more questions than it answers. How is TSA representation divided between the Aire-expressing MHC-II^high^ and mimetic compartments? What fraction of Aire-expressing cells survive to a mimetic state, how long is Aire expressed, and how is its expression silenced in such cells? Do mimetic cells feature unique attributes in comparison to their peripheral counterparts to execute thymus-specific functions? A capacity for antigen presentation by surface MHC-II appears to be at least one such difference (16). Unexpectedly, compelling outstanding questions about Aire biology now intersect with molecular developmental biology as much as with molecular genetics or cellular immunology. However, with respect to the latter, terminal differentiation of heterogeneous mimetic populations implies epithelial functions beyond antigen coverage. How mimetic subsets are organized to form functional niches, and how these shape the latest stages of lymphocyte development or contribute to overall medullary homeostasis, is an area ripe for further investigation. Indeed, exciting research has emerged supporting a functional role for neuroendocrine-like and microfold-like mimetic cells, with the latter recruiting and organizing medullary B cells in a manner mirroring peripheral Peyer’s patches (87).

In summary, time and technology have revealed that early ideas about TEC differentiation and the conceptual frameworks proposed more than 20 years ago deserve real acknowledgment (75). At the same time, Aire-expressing mTECs are clearly the source of the highest transcriptomic content and diversity within the medullary epithelium. Thus, a hybrid picture is emerging in which elements of both models are correct—Aire-expressing mTECs are not only uniquely rich sources of antigenic diversity themselves but also precursors for a diverse range of differentiated cell types, the roles and relevance of which are still being actively uncovered.

## 4. MOLECULAR MECHANISMS OF AIRE IN PROMISCUOUS GENE EXPRESSION

### 4.1. History of Medullary Thymic Epithelial Cells and Aire

Seminal studies from the Kyewski group (11), the Hanahan group (91), and others showed that mTECs have the remarkable ability to express a diverse repertoire of TSAs, a process also referred to as promiscuous gene expression (PGE), and we now know that mTECs can express up to 80–90% of the protein-coding genome (in contrast, other lineages typically express less than 65%) (15). Although mTECs were clearly implicated in PGE, the molecular mechanisms were unknown until pivotal studies showed that Aire is essential for this process (12) and is required for the expression of thousands of TSA genes (15, 92). A study of Aire-knockout mice showed that they phenocopied patients with *AIRE* mutations, developed multiorgan autoimmunity, and allowed escape of autoreactive T cells in controlled model antigen/TCR transgenic systems (93). As noted above, Aire not only mediates PGE but also appears to support the development of numerous thymic epithelial lineages (16), and any discussion of Aire’s molecular mechanism needs to recognize its potentially diverse roles beyond PGE.

How Aire functions at the molecular level has been an area of active study for more than 20 years but remains a source of active debate. Based on its structural domains, initial studies proposed that Aire may function as a transcription factor (94–96). Aire has a SAND domain that is homologous to the DNA-binding domain of Sp100 family members (97). Further studies, however, have questioned Aire’s role as a canonical transcription factor secondary to its unique properties. First, Aire-regulated genes show chromosomal clustering (98, 99). Second, these genes show stochastic expression, with individual mTECs expressing only a subset of Aire-regulated TSA genes (92, 100). Third, Aire-regulated genes vary by cell type (101). Fourth, chromatin immunoprecipitation sequencing (ChIP-seq) studies of Aire in transfected cells and mTECs have failed to identify a consensus binding motif, instead showing that Aire is located at more than 40,000 sites throughout the genome at transcriptional start sites, superenhancers, and introns (92, 102). Because of these unusual properties of Aire-mediated gene induction, there is now general consensus that Aire uses nontraditional methods for transcriptional activation. Indeed, studies over the last decade have shown that Aire multimerizes and binds to numerous proteins and that it likely acts as a general transcriptional activator to enable TSA gene expression, antigen presentation, and lineage development.

### 4.2. Aire and Promiscuous Protein Interactions

Although Aire does not appear to act as a typical transcription factor, it contains structural domains indicative of a nuclear protein. Aire contains at least five distinct functional domains, including an N-terminal CARD/HSR (homogeneously staining region), two nuclear localization sequences, two PHDs, a SAND domain, and a proline-rich region (80) (Figure 1*a*). Each of these functional domains has unique molecular functions, and deleterious human mutations in *AIRE* are scattered throughout the gene, indicating that all these domains are likely important for AIRE function. Multiple studies employing diverse methods of identifying Aire binding partners have shown that each of its structural domains is critical for facilitating various protein–protein interactions (103–106). To date, more than 40 putative binding partners have been identified and can be divided into multiple functional classes: (*a*) chromatin repression, (*b*) transcriptional elongation, (*c*) RNA splicing, (*d*) chromatin looping, and (*e*) posttranslational modification (Figure 1*b*). The following subsections discuss these classes in more detail with regard to their functions in Aire-mediated gene regulation.

#### 4.2.1. Aire interacts with proteins associated with gene repression.

As Aire is able to induce the expression of genes that would typically be silenced, it may specifically target otherwise repressed genes to allow their expression. This theory gained momentum when two early studies showed that Aire bound to the repressive histone mark H3K4me0 through its first PHD (107, 108). Moreover, histone marks of gene activation, such as H3K4me3, repress Aire’s interaction with chromatin. Importantly, patient mutations within PHD1 abrogated histone binding and mice that overexpressed *Aire* with PHD1 mutations developed autoimmunity (109). In addition, recent research has shown that Aire interacts with the repressive ATF7ip-MBD1 protein complex through its SAND domain, potentially serving as a mechanism for Aire to target genomic regions enriched in methylated DNA and the repressive H3K9me3 mark (104). Finally, studies have shown that Aire targets transcriptional start sites enriched in H3K27me3 and that deletion of a core member of the Polycomb repressive complex, EED (embryonic ectoderm development), results in alterations in TSA gene expression (15, 110). Together, these studies suggest that Aire may directly interact with both repressive histone marks and repressive protein complexes to target silent chromatin and TSA genes.

#### 4.2.2. Aire induces transcriptional elongation.

Several studies have shown that RNA polymerase II (RNAPII) is located throughout the genome; however, it is in a paused or inactive state that needs to be overcome for productive transcription (111, 112). Indeed, seminal research (113) highlighted that a key mechanism Aire uses to induce PGE is the release of stalled RNAPII from TSA gene loci to increase the efficiency of TSA gene transcription. This putative role for Aire in activating RNAPII and transcriptional elongation was supported by a study investigating TSA gene expression by using microarray analysis with mRNA-spanning probes (102). Interestingly, *Aire*-deficient mTECs were able to begin transcription at TSA genes, but full-length transcripts were not generated, indicating that Aire is required for productive transcriptional elongation. Moreover, ChIP-seq with transfected Aire showed a significant overlap of Aire and RNAPII at transcriptional start sites, further suggesting that Aire may regulate polymerase pausing to induce transcriptional elongation (102).

An important mechanism Aire uses to overcome RNAPII pausing is its interaction with the positive transcription elongation factor b (P-TEFb) (113, 114). P-TEFb exists as a heterodimer of a C-type cyclin [cyclin T1 (CycT1), CycT2, or CycK] and cyclin-dependent kinase 9 (Cdk9). It activates stalled RNAPII in part by phosphorylating the negative transcription elongation factor, causing its release from RNAPII and allowing RNAPII to begin productive elongation (115–117). One of the first Aire-binding proteins to be identified was CycT1, suggesting that Aire may utilize P-TEFb to induce transcriptional elongation (113).

An alternate mechanism by which Aire can interact with P-TEFb was found in a study showing that Aire interacts with bromodomain-containing protein 4 (BRD4) (118); however, other studies have questioned the significance of this interaction (119). The potential importance of the Aire:BRD4 interaction stems from prior studies (120–122) showing that BRD4 interacts with P-TEFb and that the interaction results in a conformational change in P-TEFb that in turn enhances P-TEFb’s kinase activity. The significance of the interaction between Aire and P-TEFb is further supported by evidence from multiple in vivo mouse models and APS1 patients. First, mice with deletion of CycT1 had loss of TSA gene expression in mTECs and developed multiorgan autoimmunity (113). Second, mice treated with BRD4 inhibitors developed autoimmunity (118). Third, a patient-derived APS1 mutation that resulted in a C-terminal truncation of AIRE abrogated the AIRE:P-TEFb interaction (123). Fourth, APS1 mutations that block acetylation of CARD blocked the binding of Aire to BRD4 (118). In summary, these studies show the importance of Aire’s interaction with P-TEFb to overcome RNAPII pausing for the induction of TSA gene expression.

Another putative mechanism by which Aire may enhance transcriptional elongation is through its interaction with DNA topoisomerases (TOP1, TOP2a, and TOP2b) and DNA damage repair proteins (DNA-PK, PARP-1, Ku70, and Ku80) (103, 124). Recent studies have suggested important functions for both DNA topoisomerases and the DNA damage repair pathways in transcription by inducing breaks in DNA to reduce DNA torsional strain and facilitate elongation (125–127). Knockdown of either DNA topoisomerases or DNA damage repair proteins in vitro reduces Aire-induced gene expression. Moreover, ChIP-seq studies in mTECs have shown that Aire localizes to superenhancers, which are known to be enriched in TOP1 (124). The importance of the Aire:TOP1 interaction in vivo is highlighted by the finding of reduced of TSA gene expression and autoimmunity in mice treated with the TOP1 inhibitor topotecan; however, these results are complicated by the diverse effects observed during treatment with such a potent systemic chemotherapeutic agent (124). Altogether, diverse recent evidence has lent support to the idea that Aire is critical for inducing transcriptional elongation through its interaction with multiple protein complexes.

#### 4.2.3. Aire enhances mRNA splicing.

Coimmunoprecipitation of Aire followed by mass spectrometry indicated that many proteins known to be involved in pre-mRNA processing bind to Aire (103, 114). These factors included RNA helicases (DDX5 and DDX17) and proteins important for RNA splicing. siRNA-mediated deletion of the identified proteins showed that they are important in Aire-induced gene expression. Functional studies indicated that Aire increases the efficiency of mRNA splicing, suggesting the mechanistic relevance of Aire’s interaction with these factors. Together, these studies indicate that Aire interacts with numerous proteins known to regulate RNA splicing, suggesting that Aire may enhance this process to further induce TSA gene expression.

#### 4.2.4. Aire interacts with proteins that affect chromatin structure.

A recent study employing high-resolution chromosome conformation capture on mTECs showed that loss of Aire induces significant changes in chromatin architecture (128). To mediate these changes, Aire binds to members of the cohesion complex (SMC1, SMC3, RAD21, and SA-2). Aire’s interaction with cohesion augments enhancer-to-promoter looping, potentially explaining some of the nuances of Aire-induced gene expression. The in vivo relevance of Aire’s interaction with cohesion was confirmed after deletion of *Stag2* (gene encoding SA-2) reduced TSA gene expression in mTECs, although it did not completely recapitulate the effect of *Aire* deletion.

#### 4.2.5. Aire is posttranslationally modified.

Early research identified a role for DNA-PK in phosphorylating the CARD of Aire (106) and found that the transcriptional coactivators CREB-binding protein and p300 acetylate Aire (129–131). Acetylation of the CARD is thought to be critical for Aire’s interaction with BRD4 (118). Interestingly, Aire also interacts with the histone deacetylase Sirtuin-1 (Sirt1), and deletion of Sirt1 in TECs causes a loss of TSA gene expression and organ-specific autoimmunity (132).Why and how Aire requires both acetylation and deacetylation for optimal function are not understood but may relate to specific lysine residues acted upon for posttranslational modification. This is an essential topic for future study.

In addition to the well-described Aire-binding proteins, a number of chromatin-remodeling complexes that are critical for PGE in TECs have been identified. These include the nucleosome remodeling and deacetylation (NuRD) complex (133), the Brahma-associated factor (BAF) ATP-dependent remodeling complex (134), and the histone acetyltransferase KAT7 (135). While they have not been shown to directly bind to Aire, their deletion in mice is associated with a reduction in TSA gene expression and autoimmunity, suggesting their importance for proper TSA gene expression. All three of these complexes (NuRD, BAF, and KAT7) are thought to open chromatin, which may be required to shape the chromatin landscape in mTECs prior to the induction of TSA expression. Provocatively, a recent study suggested that Aire may repress chromatin after BAF facilitates open chromatin in mTECs. These conclusions were based on bulk ATAC-seq data, which showed an increased number of open chromatin regions at sites targeted by Aire in Aire-deficient versus wild-type mTECs. More recent research using single-cell ATAC-seq has called into question these conclusions and demonstrated that, at single-cell resolution, Aire increases chromatin accessibility at its target genes, a conclusion that more closely aligns with its known effects on PGE (16). Nevertheless, it is clear that chromatin remodeling complexes are critical for TSA gene expression and that they likely work in concert with Aire to modulate PGE. Future studies will aim to identify additional chromatin-modeling complexes critical for Aire-dependent TSA gene expression.

### 4.3. A Model of Aire Function

Pioneering studies over the last 20 years have identified a large number of Aire binding partners involved in diverse cellular processes. The study of these binding partners, along with observations of the nuances of Aire-mediated gene regulation, suggests that Aire does not function as a sequence-specific transcription factor but rather as a general transcriptional activator to modulate PGE. Aire is localized to the nucleus and likely utilizes repressive histone marks or repressive chromatin complexes to target TSA genes. After targeting repressed chromatin, Aire activates transcriptional elongation through recruitment of P-TEFb and DNA topoisomerases and augments mRNA processing to amplify TSA gene expression. Secondary to the large number of proteins Aire interacts with and its intrinsic ability to oligomerize, it localizes to large macromolecular complexes (136–138). Interestingly, the CARD of Aire is required for homodimerization and APS1 mutations that block oligomerization have been identified, suggesting that multimerization is critical for Aire’s function (96, 136, 139). Thus, Aire seems to generate large, multimeric transcriptional activating complexes with the ability to broadly affect large portions of the genome. Future studies with increasingly sophisticated single-cell genomic techniques will hopefully facilitate an even higher resolution view of its function and role. In addition, the identification of other Aire-expressing populations presents intriguing opportunities to study its comparative function in diverse contexts.

## 5. EXTRATHYMIC AIRE-EXPRESSING CELLS AND INSIGHTS INTO SHARED BIOLOGY

While the role of Aire in central tolerance is well established, it is notable that, even from the initial positional cloning, the gene was found to be expressed not only in the thymus but also in the secondary lymphoid organs (4, 6). However, the identity, biology, and function of these populations have remained enigmatic. Recent research has generated renewed interest in the fascinating biology of these eTACs, though numerous essential questions remain.

Early mapping experiments in the Aire-knockout model with bone marrow chimeras suggested that the phenotype associated with loss of Aire is largely a central one (12). Additional data in support of this hypothesis came from Aire-knockout thymic transplant experiments into nude (*FoxN1*^−/−^) mice, which again showed autoimmunity mapping to the thymic stromal compartment (140). Notably, however, both of these approaches have limitations, as Aire has been demonstrated to be critically important in early life (141), and the nature of both bone marrow chimera and thymic-swap experiments is such that loss of extrathymic Aire in those models does not happen until adulthood.

Transcriptional Aire reporter mice have been used to define discrete populations of eTACs, which have potent tolerogenic properties and are capable of inducing deletion of cognate CD8^+^ T cells (142). Reciprocal bone marrow chimera experiments subsequently demonstrated that, unlike mTECs, eTACs are hematopoietic in origin and many of them are CCR7^+^ migratory dendritic cells (143). Further research showed that eTACs can interact with CD4^+^ T cells, that these interactions lead to functional inactivation or generation of regulatory T cells (Tregs), and that these interactions can prevent T cell–mediated autoimmune diseases like type 1 diabetes in an antigen-specific fashion (143). Conversely, the use of an AireDTR and thymic-swap system in the context of pregnancy to specifically delete eTACs demonstrated that these populations play critical roles in the maintenance of maternal/fetal immune tolerance during pregnancy (144). Indeed, loss of eTACs during only the first 9 days of pregnancy leads to adaptive immune-dependent fetal resorption and intrauterine growth restriction characterized by expansion of T follicular helper (Tfh) and Th17 populations and loss of Tregs, as well as uterine infiltration of adaptive immune cells (144).

Interestingly, migratory dendritic cells in general have tolerogenic properties (145, 146) in the context of both autoimmunity and tumor immunology (147), which appears convergent with the acquisition of Aire expression. Indeed, overexpression of Aire alone in dendritic cells has been suggested to confer tolerogenic properties; the use of either in vivo transgenic approaches using the CD11c promoter or in vitro methods via transfection and adoptive transfer of Aire^+^ dendritic cells delays the onset of type 1 diabetes in NOD mice (148, 149), and loss of Aire has been suggested to induce a proinflammatory program in dendritic cells and lead to expansion of Tfh responses and exaggerated germinal center reactions (150). Aire has recently been suggested to mediate this process via modulation of Toll-like receptor 7/8 signals and induction of perforin expression in dendritic cells (151), though all these results await rigorous in vivo corroboration with more precise conditional knockout systems.

Regarding Aire’s potential role regulating TSA expression in eTACs, the data are mixed. Microarray data from eTACs sorted from Aire wild-type and knockout eTACs initially suggested that Aire may regulate the expression of distinct eTAC-specific TSAs not shared with the thymus (142), although both the precise role of Aire and the enrichment of TSAs in these populations remain controversial. Subsequent bulk transcriptomics of Aire-sufficient and -deficient eTACs in mice identified a range of Aire-regulated transcripts that, again, are distinct from Aire-regulated genes in the thymus but are not enriched for TSAs and have unknown physiologic significance (152). A similar analysis of the Aire-dependent eTAC transcriptome was recently performed in the setting of heat-killed *Candida albicans* immunization; this study suggested that, at least in this inflammatory context, Aire regulated the expression of a range of genes involved in antigen processing and presentation (153). This idea is not inconsistent with prior results from studies of Aire-expressing B cells in the thymus, which used mixed-chimera systems to show that Aire-deficient B cells had defects in antigen presentation and maturation (154). Conversely, other recent data suggest that while Aire expression is associated with a wide array of transcriptional changes in dendritic cells, loss of Aire has a minimal impact on dendritic cells both in vitro and in vivo in terms of their transcriptomes and their ability to induce functional T cell responses in vitro (155). Notably, all these data were derived from whole-mouse Aire knockout models; therefore, the contributory effects of loss of systemic Aire cannot be discounted. Fortunately, the recent development of conditional knockout models has begun to facilitate this process (156), and further studies using such precise approaches in combination with high-resolution genomic and phenotypic characterization may be required to more fully characterize the role of Aire itself in peripheral antigen-presenting cell (APC) populations.

Human eTACs have also been identified in the secondary lymphoid organs, both by Aire protein staining (143, 157) and by single-cell transcriptomics (158, 159), and they seem to mirror their murine counterparts. Originally described as CD11c^+^, CCR7^+^, CD40^+^, and CD83^+^ by immunohistochemistry (157), these populations were subsequently found to have Aire protein in characteristic nuclear speckles by immunofluorescence (143). More recently, mass cytometry and scRNA-seq approaches identified Aire expression in CCR7^+^CD127^+^ populations, again confirming the migratory dendritic cell–like character of these populations in human secondary lymphoid organs—but, notably, these approaches did not find enrichment for TSAs in these populations (158). Similar results were found in single-cell transcriptomics, performed as part of the Human Cell Atlas cross-tissue immune cell analysis, and validated by in situ hybridization, which showed AIRE expression in human migratory dendritic cells characterized by coexpression of PDLIM1 and EBI3 (159).

Despite the accumulating body of evidence supporting the finding that many Aire-expressing cells in the periphery are largely migratory dendritic cells, recent advances have led to the identification of additional, novel Aire-expressing populations of significant biological interest. This discovery was facilitated by the technical achievement of detecting intracellular Aire protein in the periphery by flow cytometry, which led to the identification of a unique subset of CD11c^low^ eTACs characterized by high levels of expression of both Aire and the transcription factor RORγt. RORγt is otherwise known to play critical roles in the development of Th17 cells and type 3 ILCs (ILC3s) to coordinate type 3 immune responses (152). The novel RORγt^+^ eTAC subset, initially referred to as Aire^+^ ILC-like cells, have antigen processing and presentation abilities, depend on RORγt and RANK signals for their development, and share some phenotypic features with ILCs (152). However, the precise identity of this population, and its relationship to other eTAC populations, remains unclear.

To better define the identity of eTAC populations in unbiased fashion, researchers have used single-cell multiomic approaches in Aire reporter mice to shed new light on the biology of eTACs (160). These results provided the first atlas of Aire-expressing cells in the periphery and largely supported and expanded on prior conclusions—most notably that most Aire-expressing cells in the periphery are migratory dendritic cells, while smaller eTAC populations coexpressing Aire and RORγt also exist. This study has two important limitations. First, B cells were depleted prior to single-cell analysis; given the known expression of Aire in some B cell populations (154), Aire^+^ B cells may be underrepresented. Second, the study was performed in adult mice; thus, Aire^+^ populations in early and late life may be distinct.

This single-cell multiomic atlas of eTACs also revealed several key findings, including that this CD11c^low^Aire- and RORγt-coexpressing population—referred to as Janus cells—had higher transcriptional and genomic similarity to migratory dendritic cells than to ILCs and, remarkably, shared a unique transcriptional homology with mTECs. Indeed, the top differentially expressed genes in JCs were shared with only one other immune population: Aire-expressing mTECs. This transcriptional symmetry between Janus cells (a hematopoietic, mesoderm-derived population) and Aire-expressing mTECs (a stromal, endoderm-derived population) may prove to be of significant biological interest, as this shared program—consisting of a range of largely non-Aire-regulated genes—could provide novel insight into pathways critical to Aire expression and to central and peripheral immune tolerance. Interestingly, this analysis also suggested that Janus cells are enriched for a range of otherwise tissue-specific transcripts—many of which had been described as Aire regulated in eTACs (142)—though the physiological relevance of this phenomenon remains to be determined. Finally, this study suggested that not only Janus cells but also Aire-expressing migratory dendritic cells share transcriptional homology with mTECs, suggesting that considering eTACs as a group of migratory, Aire-expressing, mTEC-like, hematopoietic APCs may be a biologically relevant categorization. Though the lineage relationship of Janus cells and migratory dendritic cells remains unclear, the use of an RORγt lineage tracer demonstrated that Janus cells are not precursors to the majority of Aire^+^ migratory dendritic cells (160).

The identification of this novel Aire- and RORγt-expressing eTAC subset has generated significant recent interest, in particular because a range of significant immune-regulatory phenotypes have been associated with conditional loss of MHC-II expression in RORγt lineage APCs. Indeed a substantial body of research over the past decade has shown that loss of MHC-II in RORγt lineage cells leads to disruptions in immune homeostasis, particularly in the gut, and these phenotypes were attributed to MHC-II^+^ ILC3s (161, 162). RORγt-Cre-mediated conditional ablation of MHC-II expression results in expansion of Th1/17 CD4^+^ T cell populations in the large intestine as well as the onset of chronic inflammation characteristic of human inflammatory bowel disease.

The discovery of a potentially novel Aire- and RORγt-expressing population has thus expanded the family of RORγt lineage APCs potentially responsible for the phenotypes associated with these conditional knockout models. Recently, the question of the precise RORγt lineage cell responsible for commensal tolerance in the developing gut gained further attention in a series of three back-to-back publications (163–165) showing that RORγt lineage CCR7^+^ migratory APCs are required for the development of peripherally derived commensal-specific Tregs in the gut, which are themselves also characterized by the expression of RORγt. These three publications also identified non-ILC3 Aire^+^/RORγt^+^ populations, though they utilized distinct names for them—Janus cells (163), Thetis cells (164), and RORγt^+^ eTACs (165). For simplicity, we use the term Janus cells to refer to these Aire/RORγt^+^ populations, although we acknowledge that the nomenclature is actively evolving in this area and that subsets of these cells express varying levels of both Aire and RORγt. The literature disagrees as to which population is required for peripheral Treg induction and gut commensal tolerance—MHC-II^+^ ILC3s or Janus cells—and adjudicating the nuances of this debate is beyond the scope of this review but has been addressed elsewhere (166). Broadly, while some data support the idea that a non-Aire-expressing APC is responsible (AireDTR, AireCreER × MHC-II–flox experiments), others suggest that a non-ILC3 population is required (IL-22–Cre × MHC-II–flox, RORα–MHC-II–flox), but each approach has its own technical limitations and awaits more precise tools.

**Figure 3:**
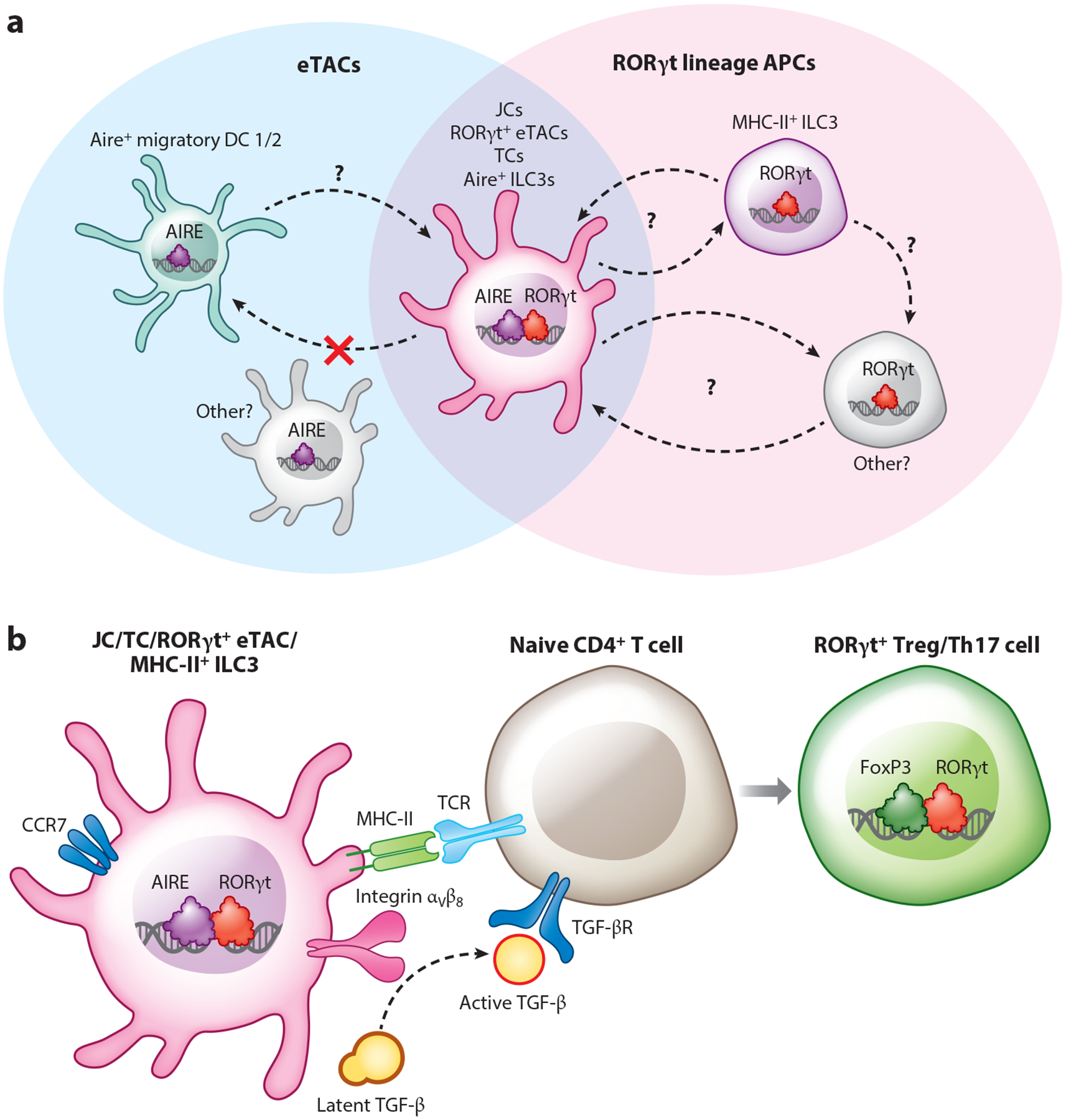
Identities and functions of eTACs. (*a*) Schematic illustration of overlap between eTACs (including Aire^+^ migratory DCs, JCs, and potentially additional populations present in early life; in various disease contexts; and in B cell lineages) and RORγt lineage APCs. The precise identities and lineage relationships between these populations remain largely undefined, though JCs are not precursors for most Aire^+^ migratory DCs. (*b*) Potential mechanism of RORγt^+^ Treg induction by RORγt lineage APCs, which depends on CCR7-mediated migration, MHC/TCR interactions, and activation of latent *TGFB* via cell surface integrin α_V_β_8_. Abbreviations: APC, antigen-presenting cell; DC, dendritic cell; eTAC, extrathymic Aire-expressing cell; JC, Janus cell; TC, Thetis cell; TCR, T cell receptor; Th17, T helper 17; Treg, regulatory T cell.

Thus, the lineage relationship and functional contributions of Janus cells, ILCs, and migratory dendritic cells remain a source of active debate, as do the relative contribution of each population to commensal-specific peripheral Treg induction and even the nomenclature of this population (or populations) and the identity of any subsets (Figure 3). It is clear, however, that Janus cells are enriched in early life and potentially in the mesenteric lymph nodes (152, 164) and that the mechanism of commensal-specific peripheral Treg induction appears to require RORγt lineage CCR7-dependent cell migration, antigen presentation via MHC-II, and activation of latent TGF-β via cell surface integrin α_V_β_8_ (163–165). It is tantalizing to speculate about parallel roles for Aire-expressing populations in the early-life induction of immune tolerance to not only self but also commensal “meta-self, ” but more careful investigation is required to precisely identify the responsible populations.

Despite the potential roles of Janus cells in commensal tolerance, recent evidence has also demonstrated a surprising result in mice conditionally deficient in Aire in RORγt lineage populations: that loss of Aire in a repeat-immunization model using heat-killed *C. albicans* leads to significantly impaired type 17 immune responses in this context (153). Given that *C. albicans* is not a commensal organism in mouse and that these experimental systems require numerous repeat immunizations to elicit the phenotype, it remains unclear what bearing this result has on its role in Janus cells during normal physiologic conditions; however, it represents a distinct phenotype associated with the conditional loss of Aire in Janus cells. Furthermore, this finding suggests that the function of Aire in these populations may be context dependent, as is true for many other immune populations. As in the thymus, Aire in the periphery may play a role in facilitating antigen processing and presentation—in this case, perhaps, to not only self but also meta-self and commensal organisms—the outcome of which varies according to context.

Thus, although the study of Aire and Aire-expressing populations outside the thymus is in its infancy, it has already yielded a range of novel insights into immune homeostasis that we are only beginning to explore. It will be of great interest to determine the parallels between Aire-expressing populations in the thymus and periphery to define novel biology through both their similarities and differences, including novel regulators and regulatory regions governing Aire expression, the roles of Aire itself in these respective populations, the lineage and the essential biology and function(s) of Janus cells, and the potential roles of these Aire-expressing APC populations in a range of clinically relevant contexts ranging from tumor immunity to transplantation to autoimmunity. Furthermore, and parallel to recent discoveries in the thymus, it will be interesting to determine the identity of any post-Aire eTAC populations. More broadly, understanding the shared and divergent biology of Aire-expressing cells in the thymus and periphery may yield novel insights into core regulatory mechanisms of immune tolerance.

## 6. CONCLUSIONS

Although the identification of *AIRE* as the causative gene in APS1 and the discoveries about its role in central tolerance happened more than 20 years ago, the study of Aire and Aire-expressing populations continues to reveal surprising new insights into immune tolerance, autoimmunity, and human disease. The past few years have witnessed a surge in exciting, innovative, and still-unfolding biology on several fronts. Notably, the use of advanced phage-display technologies is allowing for unprecedented levels of insight into the autoantigens targeted in the absence of Aire, with diverse implications for diseases from lipodystrophy to severe COVID. The recent discovery of the panoply of post-Aire thymic epithelial mimetic populations, and the role of Aire in this developmental process, is an area of active study with the potential to yield insights extending beyond the thymus to change our basic understanding of cellular plasticity and cell fate determination. Ultimately, defining the biology of these lineages will require further untangling of the molecular mechanism of Aire, which will surely inform its emerging dual roles in both cellular differentiation and broad transcriptomic coverage for antigen presentation. The biology of eTAC populations has dramatically advanced our understanding of both the identity and function of these fascinating populations. Insights include the demonstration that eTACs are required for normal immune tolerance during pregnancy, the identification of novel Aire/RORγt^+^ Janus cells and related populations, and the discovery of their putative roles in microbial tolerance and immunity. Finally, identification of a remarkable core transcriptional symmetry between Aire-expressing cells in the thymus and in the periphery is striking and may yield diverse and innovative new avenues for inquiry into fundamental mechanisms of immune homeostasis.

## AIRE AND APS1 OFFER KEY INSIGHTS INTO SEVERE COVID-19 SUSCEPTIBILITY

During the early stages of the COVID-19 pandemic, one of the more vexing questions was: Why do certain adults develop more severe disease than others? Clinicians caring for APS1 patients with COVID-19 noted that several developed severe pneumonia (33, 34). APS1 patients frequently harbor neutralizing autoantibodies to type 1 interferons, particularly type 1 IFN-α and -ω family members (23). In light of the key role of type 1 interferons in the early response to viral infection, investigators tested the hypothesis that a significant fraction of patients with severe COVID-19 harbored such anti-interferon autoantibodies. Indeed, an initial analysis found that as many as 10% to 15% of patients with severe COVID-19 had neutralizing anti-interferon autoantibodies (33). These data have now been replicated by multiple groups, and there is clear consensus that these antibodies represent an established mechanism underlying susceptibility to severe COVID-19 (35). Later studies with larger numbers of APS1 subjects also demonstrated this association and showed that interferon autoantibodies predated infection (36).

Interestingly, studies in the general population have shown that these same neutralizing autoantibodies are detectable in approximately 0.3% of adults, but this number rises significantly (to 3–4%) in adults older than 70 years (37–39). Clearly, most of these patients do not harbor functional mutations in *AIRE* or have APS1, leading to an interesting question: What other factors predispose to these autoantibodies? Other known associations point to defects in the thymus, as patients with a history of thymoma frequently develop such autoantibodies (40) and *AIRE* expression is frequently defective in thymomas (41, 42). Other rare genetic mutations that are associated with these autoantibodies involve genes that regulate *AIRE* expression, including C-terminal mutations in *NFKB2* (43). Mutations in the X-linked gene regulator *NEMO*, hypomorphic *RAG1* and *RAG2* mutations, and mutations in *FOXP3* have also been implicated (33, 44, 45). These associations suggest that defects in proper thymic development and selection may predispose patients to the development of anti-interferon autoantibodies. Indeed, the presence of neutralizing autoantibodies to type 1 interferons is the second-highest risk factor for severe COVID-19 (after age), underscoring the importance of these insights (37).
